# Suicide Risks among Adolescents and Young Adults in Rural China

**DOI:** 10.3390/ijerph120100131

**Published:** 2014-12-23

**Authors:** Sibo Zhao, Jie Zhang

**Affiliations:** 1 School of Social Development, Central University of Finance and Economics, Beijing 100081, China; 2 Department of Sociology, State University of New York at Buffalo, Buffalo 14260, USA; E-Mail: sibozhao@buffalo.edu; 3 Department of Sociology, State University of New York College at Buffalo, Buffalo 14222, USA

**Keywords:** suicide, risk factors, adolescent, young adult

## Abstract

*Background*: In China, suicide is one of the major causes of death among adolescents and young adults aged 15 to 34 years. *Aim*: The current study examines how risk factors vary by age groups in rural China, referring to those aged 15 to 24 years and those aged 25 to 34 years. *Method*: A case-control psychological autopsy (PA) study is conducted in sixteen counties from three Chinese provinces, including 392 suicide cases and 416 community living controls in the sample. *Results*: In China, young adults aged 25 to 34 years have a higher risk for suicide than adolescents aged 15 to 24 years, and it holds true even controlling for relevant social factors. In addition, age-related factors such as education, marital status, whether having children, status in the family, physical health, and personal income all have varying degrees of impact on suicide risks for rural youth. *Conclusions*: This study shows that there are some age-related risk factors for suicide at certain life stages and emphasizes that young adults in rural China aged 25 to 34 years have an increased risk of suicide as a result of experiencing more psychological strains with age.

## 1. Introduction

Suicide prevention is one of the World Health Organization’s priorities in mental health for developing countries. According to data on suicide rates in 2002–2011 provided by the Chinese Ministry of Health (MOH) and the National Population Census (NPC), the total number of suicide deaths was roughly 200,000 every year. The estimated mean national suicide mortality rate was 8.3 per 100,000 people each year in the most recent six years [1]. Even with a decrease of the suicide rates observed in China in the past decade [2,3], suicide has been identified as the number one cause of death in the country for those between 15 and 34 years of age [4]. Therefore, more empirical evidence is needed to advance our understanding of suicidal youth in the Chinese population to better understand how risk factors for suicide are related to different age groups. Only by recognizing those who are at risk for suicide and knowing how to provide treatment for suicidal individuals can improve how we deal with this urgent issue.

As Durkheim noted at the end of the nineteenth century, suicide rates increased with age. The lowest rates appeared among the young and the highest among the elderly [5]. However, this pattern has changed substantially in recent years. Researchers have documented that age trends in suicide rates for the United States follow a certain pattern that the incidence of suicide is sharp increased from youth (at their early twenties) to adulthood and, slightly decreased to a lower rate throughout middle age [6]. In recent years, suicide has become the third leading cause of death among persons aged 15 to 24 years, and the second among persons aged 25 to 34 years [7].

Youth suicide, as an especially significant aspect in the suicide literature, has drawn much attention in recent years [8,9,10]. Some studies focus on how mental health risk factors impact on youth suicidal behaviors [11]. It suggests that psychopathological symptoms are associated with suicidal behavior. Particularly, more than 90% of youth suicide victims have had at least one major psychiatric disorder, although younger adolescent (<16 years) suicide victims have lower rates of psychopathology, averaging around 60% [12,13]. Substance abuse is more common and leads to a much higher risk for suicide in the older (> or =16 years) *vs.* younger (<16 years) adolescents [12]. Depressive disorders (major depression, depressive disorders not otherwise specified, bipolar disorders, dysthymia) are consistently the most prevalent disorders among adolescent suicide victims, ranging from 49% to 64% of cases [14]. Other scholars pay more attention on the effect of sociodemographic factors [8], arguing the importance of childhood adversities, family characteristics, and socioeconomic status on studying suicide risks among young people [15,16,17]. For example, focusing on the importance of considering how social context impact age-related trajectories in suicide and homicide in high-income nations, Pampel and Williamson [18] demonstrate that increases in youth suicide rates are associated with increases in the size of the youngest (15–24 years of age) cohort, as well as recent changes in traditional family roles and family stability.

Unlike Western studies on suicidal youth, previous studies with Chinese data have documented some unique characteristics of Chinese rural young suicide victims. For example, scholars have argued that the suicide rate in women is higher than in men [19,20]; marriage does not play a protective role for suicide in rural China [21] and neither does religion [22]. In addition, other correlates, for example, multiple psychosocial factors and life stress are reported to be the risk factors for suicidal behaviors [23]. However, study on the suicide risk with age group comparisons is rare in China. This study is designed to test how suicide risks are different between adolescents aged 15 to 24 years and young adults aged 25 to 34 years in rural China. Several factors associated with individual age may potentially have influences on the correlation observed between age and suicide risk according to the previous studies [12]. In particular, those factors that included in the current study are education, marital status, whether having children, status in the family, physical health, and personal income.

In the suicide literature, several theories are commonly used to explain the mechanisms behind the suicidal behaviors among different social groups. One of the pioneers of sociology, Durkheim [5] showed in his classic study of suicide that it was more prevalent among individuals who were not married or who had no involvement in the church or community. His explanation was that individuals who were not well integrated into society had little social support, and, therefore, were at increased risk of suicide. To move beyond the traditional approach of using social integration as the dominant structural consideration to study suicide, a growing body of recent studies has used the strain theory of suicide to guide research [24,25]. According to the strain theory of suicide, suicide as well as mental disorders, can be preceded by psychological strains in the social structure [25]. A psychological strain is formed by at least two stressors [26,27], conflicting in an individual’s life and leading to different directions, so that the individual will feel helpless, hopeless, and in a psychologically torturous situation [28]. For the strain theory of suicide, any of the four types of strains will increase individuals’ suicide risk, and those strains result from differential values, discrepancy between aspiration and reality, relative deprivation, and lack of coping skills in a crisis. The connection between suicide and psychological strains in the form of all four sources has been tested and supported with a sample of suicide notes in the United States [28] and through psychological autopsy studies in rural China [25]. In the current study, we use Chinese rural youth suicide data to illustrate how age and age-related factors impact suicidal behavior. We also try to use strain theory of suicide to better understand these differences between adolescents and young adults in order to provide effective suicide prevention strategies and treatment programs for certain at-risk population.

## 2. Methods

### 2.1. Subjects and Data Collection

Data for the study were obtained from sixteen rural counties in three Chinese provinces (Liaoning, Hunan, and Shandong). It was a large psychological autopsy project investigating correlates of completed suicide in comparison with a group of living controls. In each of the 16 counties, suicides aged 15–34 years were sampled consecutively from October 2005 to June 2008. Similar numbers of community-living controls were recruited in the same counties for the same time periods. In this study, we have excluded cases of accidental or natural death in which suicidal intent was questioned. As China does not have a medical examiner system and all deaths are required to be sent to a health agency for a death certificate, hospitals are the primary place for the Centers for Disease Control and Prevention (CDC) to locate cases for the study. Each hospital uses a standard protocol to determine the cause of death. In remote rural areas far away from a hospital, village doctors are responsible for furnishing death certificates and are required to report the death to the *Xiang* (township) health agency. All the hospitals and clinics in the county are supervised by the county CDC. For our study, all suicidal deaths were required to be reported to each county CDC by telephone or fax on a daily basis, and the information gathered at the county CDC was then forwarded on a monthly basis to the provincial CDC. For those suicidal deaths that were not recognized by any health agency, our mortality registry system allowed the village treasurers, who collect fees for each burial or cremation and are aware of all the deaths in the village, to notify the *Xiang* health agency or the county CDC. Whenever necessary, an investigation with the village board and villagers was conducted by the research team to try to ensure that no suicide cases were missed, or reported erroneously. These procedures were implemented to minimize false classifications.

The community-living controls were from the same counties and from among the living general population within the same age group of the suicides. In each province, we used the 2005 census database of the counties in our research. For each suicide, we used the database of the county where the deceased lived to randomly select a living control in the same age range (*i.e.*, 15–34 years). With regard to gender, the random selection of controls aged 15–34 years from each county database yielded approximately equal numbers of males and females, which approximated to the gender distribution of suicide cases in the study. The control sample did not exclude individuals who had been diagnosed with mental disorders or previous suicide attempts. Thus, the prevalence of mental disorders and suicidal attempts could be assessed in the rural general population aged 15–34, and more importantly, the effects (direct, moderating and intervening) of mental disorders on completed suicide could also be studied.

Face-to-face interviews were conducted in households in villages. In each selected county, suicide cases and living controls were enrolled and two informants (one family member and one friend or neighbor) were interviewed for each suicide case or control. The controls themselves were also interviewed with the same protocol to obtained further information of the study. As a result, 392 suicide cases and 416 community living controls were recruited in this study. The study protocol was approved by the Institutional Review Board at the State University of New York College at Buffalo, as well as by the three universities in China where the data collection was conducted. For the specific procedures in sampling and interviewing and our protocol for the human subjects protection, please refer to our previous publications [21,29]. Table 1 presents the demographic characteristics of the sample, as well as the major independent and dependent variables for the study with age groups comparisons.

### 2.2. Measures

The dependent variable is a dichotomous variable in which 0 represents a higher risk of death and 1 represents a lower risk of death. As mentioned above, suicide is a major social and public health problem in China. Thus, not only individual factors, but also a range of social factors are considered in attempts to explain suicidal behavior. Those predicting variables include gender, marital status, education, status in the family, whether having children, physical health, personal income and depression. Age ranged from 15 to 34 years, which was divided into two groups for further comparison. The two groups are adolescents aged 15 to 24 years and young adults aged 25 to 34 years.

**Table 1:** Descriptions of the sample for the major variables and their age group comparisons.

Variable	All Subjects (N = 808)						Suicide Cases (N = 392)						Controls (N = 416)					
	Adolescents		Young Adults		Comparison Statistcs^#^		Adolescents		Young Adults		Comparison Statistcs ^#^		Adolescents		Young Adults		Comparison Statistcs ^#^	
	Mean or f	SD or %	Mean or f	SD or %	*t/χ^2^*	*p*	Mean or f	SD or %	Mean or f	SD or %	*t/χ^2^*	*p*	Mean or f	SD or %	Mean or f	SD or %	*t/χ^2^*	*p*
*Gender*					0.304	0.617					0.042	0.916					1.201	0.273
Women	149	47.30%	243	49.30%			65	46.10%	113	45.00%			84	48.30%	130	53.70%		
Men	166	52.70%	250	50.70%			76	53.90%	138	55.00%			90	51.70%	112	46.30%		
*Marital status*					298.82	<0.001					111.522	<0.001					202.695	<0.001
Not currently married	243	77.40%	75	15.80%			114	80.90%	59	24.90%			129	74.60%	16	6.70%		
Currently married	71	22.60%	401	84.20%			27	19.10%	178	75.10%			44	25.40%	223	93.30%		
*Education*	8.45	2.535	8.19	2.845	1.299	0.194	7.57	2.859	7.28	2.715	0.97	0.333	9.16	1.976	9.13	2.668	0.115	0.908
*Status in the family*					10.117	0.006					8.053	0.018					14.216	0.001
Low	21	6.70%	42	8.60%			14	9.90%	42	16.80%			7	4.00%	0	0.00%		
Average	284	90.20%	408	83.10%			121	85.80%	184	73.60%			163	93.70%	224	92.90%		
High	10	3.20%	41	8.40%			6	4.30%	24	9.60%			4	2.30%	17	7.10%		
*Having Children*					353.418	<0.001					145.553	<0.001					217.683	<0.001
No	272	86.30%	93	18.90%			126	89.40%	65	25.90%			146	83.90%	28	11.60%		
Yes	43	13.70%	400	81.10%			15	10.60%	186	74.10%			28	16.10%	214	88.40%		
*Physical Health*					25.608	<0.001					25.626	<0.001					2.134	0.344
Poor	24	7.60%	83	16.80%			18	12.80%	70	27.90%			6	3.40%	13	5.40%		
OK	47	14.90%	110	22.30%			18	12.80%	60	23.90%			29	16.70%	50	20.70%		
Good	244	77.50%	300	60.90%			105	74.50%	121	48.20%			139	79.90%	179	74.00%		
*Personal income*					25.757	<0.001					1.507	0.471					35.059	<0.001
Low (≤10,000)	214	70.60%	252	55.10%			97	70.30%	156	64.50%			117	70.90%	96	44.70%		
Average (10,001–19,999)	65	21.50%	113	24.70%			31	22.50%	62	25.60%			34	20.60%	51	23.70%		
High(≥20,000)	24	7.90%	92	20.10%			10	7.20%	24	9.90%			14	8.50%	68	31.60%		
*HAM-D depression*	4.63	8.522	8.2	13.128	−4.617	<0.001	10.03	10.458	16.39	14.82	−4.434	<0.001	0.29	1.056	0.42	1.84	−0.886	0.376

**#** All the significant differences are functions of the age.

The Hamilton Depression Rating Scale (HAM-D Scale) was designed to evaluate the degree of and variance in depression [30]. It quickly became the standard measure of depression severity for clinical trials of antidepressants [31]. The Hamilton depression scale has been the gold standard for the assessment of depression for 40 years and is now the most commonly used measure of depression [32]. For each of the 24 items in the full version of the scale, respondents were asked to rate the severity of their depression by probing mood, feelings of guilt, suicide ideation, insomnia, agitation or retardation, anxiety, weight loss, and somatic symptoms. The translation of the 24-item scale was borrowed from Tang [33], and has already been tested with Chinese depressive inpatients showing good reliability and validity. There were two proxy interviews for each suicide case and each living control. The vast majority of the responses for the target person were the same or fairly similar. For different responses pertaining to the target person, we selected the response representing a positive symptom, because the other informant may not have had an opportunity to observe the specific characteristic or behavior. The range of potential HAM-D scores is from 0 to 76. A higher score indicates more severe depression. In addition to considering HAM-D depression score, we examined a self-reported measure of physical health, which was categorized as “poor,” “OK,” or “good.”

To investigate the effect of marriage and marital experience on suicide risks between adolescents and young adults in rural China, we computed a variable with two categories. The group of “not currently married” included those young people who had never been married, were divorced, were separated, or were widowed. The group of “currently married” covered those who were currently married or involved in cohabitation.

For the question of “whether having children,” the respondents were asked to mark “yes” with all positive responses and “no” with all negative responses. Based on the scale, the status in the family was categorized as “low,” “average,” or “high.” Personal annual income was measured in Chinese Renminbi (RMB), which were divided into three levels based on the scale mean: low (≤10,000), average (10,000–19,999), or high (≥20,000). One U.S. dollar was equivalent to about 7.50 RMB at that time.

### 2.3. Data Analysis and Certification of the Methods

SPSS 21.0 was used for all statistical analysis. Coefficients have been converted to odds ratios (ORs) for the interpretation and 95% confidence intervals would also be reported. Both authors of this study certify that we are responsible for the methods used in the data collection and data analyses.

## 3. Results

Table 1 illustrates the distribution (mean or frequency) of each of the expected correlates of suicide risk for all subjects and their comparison between adolescents (aged 15 to 24) and young adults (aged 25 to 34). In this study, adolescents (N = 315) and young adults (N = 493) accounted for about 39% and 61% of the sample, respectively. The distribution of the respondents according to gender shows a generally similar pattern for adolescents and young adults. A total of 47.3% of women and 52.7% of men are aged 15 to 24 years for the adolescents group, while 49.3% of women and 50.7% of men are aged 25 to 34 years for the young adults group.

Duration of education ranged from 0 to 15 years for the respondents in the adolescents group and from 0 to 18 years for the respondents in the young adults group. The means of education level for the adolescents group and the young adults group were thus 8.45 years and 8.19 years. Compared to the respondents in the adolescents group, those in the young adults group are more likely to be currently married (84.2%) and have at least one child (81.1%). They also consider themselves in either lower (8.6%) or higher (8.4%) status in the family compared to adolescents, although most of young adults (83.1%) still report average in the scale. In addition, more respondents from young adults group report poorer physical health and higher personal annual income than the respondents from the adolescents group. As predicted in the literature, young adults aged 25 to 34 years are more likely to associate with higher score of depression as well (See Table 1).

T-test and Chi-square tests have been used for the comparison between the adolescents group and the young adults group on suicide risk and major variables. The results indicate that the respondents in young adults group have significant differences with those in adolescents group on marital status, status in the family, whether have children, physical health, personal income and depression level.

Bivariate analyses for adolescents (see Table 2) indicate that lower suicide risk has associated with higher education and lower depression level. The associations are statistically significant. Women tend to have higher suicide risk than men, but the data does not show any significant difference. For young adults aged 25 to 34 years (see Table 3), lower suicide risk is correlated with higher education, higher status in the family, better physical health, higher personal income, and lower depression score, all of which are statistically significant. Being married and having kids are also positively related to lower suicide risk in our sample.

**Table 2:** Bivariate Inter-correlations among Predictors and Suicide for Adolescents, 15–24 years of age (N = 315) **^#^**.

Variable	Case Control Status	Gender	Marital Status	Education	Status in the Family	Children	Physical Health	Personal Income	HAM-D
Gender	−0.044	—							
Marital Status	0.18	−0.528 **	—						
Education	0.418 **	−0.068	−0.091	—					
Status in the Family	0.204	−0.149	0.259	0.103	—				
Having Children	0.234	−0.588 **	0.988 **	−0.082	0.398	—			
Physical Health	0.187	0.089	0.203	0.070	−0.305	−0.016	—		
Personal Income	−0.005	0.237 *	0.271	−0.047	−0.011	0.031	0.304 **	—	
HAM-D Depression	−0.739 **	0.073	−0.090	−0.270 **	−0.103 *	−0.192	−0.172 **	0.023	—

**^#^** Gamma is used as measure of association; ** Correlation is significant at the 0.01 level (2-tailed); * Correlation is significant at the 0.05 level (2-tailed).

**Table 3:** Bivariate Inter-correlations among Predictors and Suicide for Young Adults, 25–34 years of age (N = 493) **^#^**.

Variable	Case Control Status	Gender	Marital Status	Education	Status in the Family	Children	Physical Health	Personal Income	HAM-D
Gender	−0.173	—							
Marital Status	0.644 **	−0.763 **	—						
Education	0.462 **	0.026	0.073	—					
Status in the Family	0.423 **	‒0.114	0.582 **	0.151	—				
Having Children	0.455 **	−0.706 **	0.971 **	−0.007	0.367 *	—			
Physical Health	0.522 **	‒0.107	0.310 **	0.103	0.109	0.013	—		
Personal Income	0.412 **	0.377 **	0.127	0.376 **	0.202 *	−0.005	0.266 **	—	
HAM-D Depression	−0.788 **	0.127 **	−0.435 **	−0.318 **	−0.337 **	−0.161 **	−0.469 **	−0.314 **	—

**^#^** Gamma is used as measure of association; ** Correlation is significant at the 0.01 level (2-tailed); * Correlation is significant at the 0.05 level (2-tailed).

Unconditional logistic regression model is used for analyzing the data of the suicide subjects to illustrate how suicide risks are different between adolescents and young adults with the relevant variables controlled for. Hosmer-Lemeshow goodness-of-fit (GOF) tests help us decide whether the model is correctly specified (see Table 4). The p-value for model 1 is below 0.05 significant level (0.033) as a result of including the variable of depression, which indicates that we have to reject the model. Model 2 (without depression) shows that, controlling for other variables, the odd ratio of adolescents not experiencing suicide risk is 2.511 times higher than the odds for young adults, which indicates that young adults are more likely to experience higher risk for suicide in our sample. For all subjects, each additional year of education is consistently associated with a lower risk for suicide. The odd ratio associated with a one year increase in education is 1.309 (95% CI 1.216 to 1.408), indicating the higher education, the lower the chance to experience suicide risk. The odd ratio of low family status respondents (0.196) indicates that individuals who feel like at low family status are more likely to have higher suicide risk and it is statistically significant at the α = 0.05 level. Compared to those who report good physical health, respondents who have poor or average health are more likely to have higher suicide risk. Compare to those respondents who have children in the family, the respondents who do not have children in the family are more likely to have higher suicide risk (OR = 0.367). In addition, individuals who have poor or average personal income show higher risk for suicide compared to those who report themselves as having a good personal income. With other variables controlled for, gender and marital status are not significant predictors in the logistic regression analyses.

**Table 4:** Logistic multiple regression predicting suicide (0 = suicide; 1 = control) with major covariates for all subjects (N = 808).

Predictor	Model 1				Model 2			
	OR	95% CI (Lower–Upper)		*p*	OR	95% CI (Lower–Upper)		*p*
Education	1.265	1.137	1.407	<0.001	1.309	1.216	1.408	<0.001
Age group								
Adolescents	1.708	0.808	3.608	0.161	2.511	1.479	4.263	0.001
Young adults	1.0 (-)	1.0 (-)	1.0 (-)		1.0 (-)	1.0 (-)	1.0 (-)	
Gender								
Women	0.692	0.416	1.152	0.157	1.011	0.711	1.438	0.950
Men	1.0 (-)	1.0 (-)	1.0 (-)		1.0 (-)	1.0 (-)	1.0 (-)	
Marital status								
Not currently married	0.763	0.308	1.893	0.560	0.772	0.417	1.43	0.411
Currently married	1.0 (-)	1.0 (-)	1.0 (-)		1.0 (-)	1.0 (-)	1.0 (-)	
Status in the family								
Low	0.621	0.138	2.801	0.535	0.196	0.065	0.594	0.004
Average	2.448	0.967	6.197	0.059	1.615	0.823	3.17	0.163
High	1.0 (-)	1.0 (-)	1.0 (-)		1.0 (-)	1.0 (-)	1.0 (-)	
Having Children								
No	0.431	0.158	1.175	0.100	0.367	0.19	0.709	0.003
Yes	1.0 (-)	1.0 (-)	1.0 (-)		1.0 (-)	1.0 (-)	1.0 (-)	
Physical Health								
Poor	0.845	0.327	2.185	0.729	0.183	0.101	0.332	<0.001
OK	1.722	0.835	3.554	0.141	0.58	0.38	0.884	0.011
Good	1.0 (-)	1.0 (-)	1.0 (-)		1.0 (-)	1.0 (-)	1.0 (-)	
Personal income								
Low (≤10,000)	0.567	0.259	1.243	0.157	0.556	0.33	0.939	0.028
Average (10,001–19,999)	0.549	0.237	1.274	0.163	0.548	0.309	0.973	0.040
High (≥20,000)	1.0 (-)	1.0 (-)	1.0 (-)		1.0 (-)	1.0 (-)	1.0 (-)	
HAM-D Depression	0.527	0.464	0.599	<0.001				
Constant	0.817			0.779	0.217			0.003
R^2^	0.719				0.317			
Hosmer-Lemeshow (χ^2^)	16.737			0.033	11.778			0.161

(-) Reference category.

Logistic regression analysis has also been used for age group comparison in the study (see Table 5). The model fit for the young adults group (R^2^ = 0.445) is better than for the adolescents group (R^2^ = 0.180). Education is a strong predictor for both age groups on suicide risk. Among adolescents, respondents who have poor physical health (OR = 0.845) are more likely to have higher suicide risk compare to those who report good physical health. However, we do not find a significant difference on suicide risk between respondents who report average physical health and good health.

In the young adult group, respondents who report a poor physical health (*p* < 0.001) and an average physical health (0.001) are found to have higher suicide risk than those who report a good physical health. In addition, “whether have children” is another significant predictor in the young adults group, indicating that people who do not have children have a higher suicide risk. People who have lower personal income also shows higher risk for suicide compared to people who have high personal income among young adults. With other variables controlled for, gender, marital status, and status in the family are not significant predictors for either age group in the logistic regression analyses.

**Table 5:** Logistic multiple regression predicting suicide (0 = suicide; 1 = control) for age group comparisons.

Predictor	Adolescents (N = 315)				Young adults (N = 493)			
	OR	95% CI (lower–upper)		*p*	OR	95% CI (lower–upper)		*p*
Education	1.321	1.174	1.486	<0.001	1.285	1.167	1.415	<0.001
Gender								
Women	0.922	0.556	1.529	0.753	1.073	0.646	1.782	0.786
Men	1.0 (-)	1.0 (-)	1.0 (-)		1.0 (-)	1.0 (-)	1.0 (-)	
Marital status								
Not currently married	0.898	0.396	2.035	0.796	0.470	0.171	1.293	0.144
Currently married	1.0 (-)	1.0 (-)	1.0 (-)		1.0 (-)	1.0 (-)	1.0 (-)	
Status in the family								
Low	0.755	0.127	4.476	0.757	-	-	-	0.997 ^#^
Average	2.313	0.537	9.964	0.260	1.723	0.779	3.810	0.179
High	1.0 (-)	1.0 (-)	1.0 (-)		1.0 (-)	1.0 (-)	1.0 (-)	
Having Children								
No	0.559	0.202	1.550	0.264	0.318	0.132	0.770	0.011
Yes	1.0 (-)	1.0 (-)	1.0 (-)		1.0 (-)	1.0 (-)	1.0 (-)	
Physical Health								
Poor	0.345	0.121	0.981	0.046	0.145	0.069	0.307	<0.001
OK	1.056	.534	2.087	0.877	0.395	0.226	0.691	0.001
Good	1.0 (-)	1.0 (-)	1.0 (-)		1.0 (-)	1.0 (-)	1.0 (-)	
Personal income								
Low(≤10,000)	0.917	0.364	2.308	0.854	0.428	0.220	0.832	0.012
Average (10,001–19,999)	0.800	0.290	2.206	0.666	0.518	0.250	1.071	0.076
High (≥20,000)	1.0 (-)	1.0 (-)	1.0 (-)		1.0 (-)	1.0 (-)	1.0 (-)	
Constant	0.122			0.042	0.341			0.090
R^2^	0.180				0.445			
Hosmer-Lemeshow (χ^2^)	4.888			0.770	6.691			0.570

**^#^** The uncertainty of parameter estimation is due to the multicollinearity in the model; (-) Reference category.

## 4. Discussion

Previous studies have focused on different risk factors between suicide group and control group [34]. This study is organized around two main goals. First, it is to find out whether age differences exist among at-risk people in rural China so that we can pay more attention on reducing suicide risks for a particular age group. Second, it should be known if certain age-related factors such as education, marital status, whether have children, status in the family, physical health, and personal income play roles in either decreasing or increasing suicide risks for adolescents and young adults in rural China that can help us have a better understanding of Chinese rural youth suicide. Our findings indicate that there is a difference between two age groups concerning suicide risks and young adults have faced more suicide risk factors than adolescents in rural China.

We have found that women in the adolescents group have higher risk for suicide than men, whereas women in the young adults group have lower risk for suicide than men. However, the gender difference does not show significant impact in our sample. Another non-significant predictor is status in the family. Although low family status is likely to lead to high suicide risk for all subjects in our model, we don’t find different effects between two age groups due to the multicollinearity as fewer cases in the young adults group. More studies are needed in the future.

According to the strain theory of suicide, the four sources of strain are: (a) differential value conflicts, (b) discrepancies between aspiration and reality, (c) relative deprivation, and (d) lack of coping skills. The differences between two age groups on suicide risks in rural China can be partially explained by the strain theory of suicide because young adults have potentially experienced more conflicting life stressors in their life than adolescents and, therefore, are more likely to produce psychological strains.

For example, our results show that education and physical health are significant predicators for suicide risk for both respondents in the adolescents group and young adults group. Education plays a key role in providing individuals with knowledge about coping skills. Individuals, including adolescents and young adults, who have higher education will have better coping skills to lower the risk for suicide in rural China. Compared to an adolescent, if an individual have entered into adulthood, both society and the individual will have certain expectations for his or her life, such as being employed or taking care of his or her family. However, if a young adult does not have a good physical health he or she may have to face the reality that he or she might fail to reach his or her aspirations. These discrepancies between his or her aspirations and reality may produce strains and, then, lead to increased risk for suicide.

Furthermore, for young adults aged 25 to 34 years, getting married and having kids are important transitions in their life stage for people in rural China. Researchers have studied the order in which life events and transitions occur and their effects on mental health. They suggest that the effects of stressful events may vary, depending on the event and the age of exposure [35,36]. In rural China, it is very rare for an individual who is older than 25 to be single or not have kids. It is a shame for the individual or even for the whole family so that values strains may occur because of these circumstances. Therefore, although the effect of marital status on suicide risk is not statistically significant between adolescents and young adults, individuals who are not currently married and do not have children among age 25 to 34 will feel more stress from both family and society, which may produce value strains and thus increase their risk for suicide.

In addition, low personal income shows a statistically significant impact on suicide risk among young adults. Stack [37] found that relative deprivation (*i.e.*, income inequality), instead of a single life stressor (*i.e.*, income crisis), would be better explanation for the risk of suicide. According to the strain theory of suicide, in the situation where a poor individual realizes that other people of the same or similar background are leading a much better life, the person may experience the deprivation strain. This might be a possible reason leading the increased risk for suicide.

In sum, the age-related factors are more observable in young adults aged 25 to 34 years than in adolescents aged 15 to 24 years in rural China. Such factors as education, physical health, whether have children and personal income should be paid more attention to in this particular population. Suicide behaviors have been identified as consequences of psychological strains, according to the theory. Therefore, we can develop suicide prevention strategies by eliminating the strains rather than removing life stress to reduce the risks of suicide. To some extent, encouraging higher education and providing job training can reduce the value strains, aspiration strain, and deprivation strain, as well as improve one’s coping strategies. The current study is limited by its data collection approach and is, therefore, not able to study how these risk factors affect Chinese rural young subjects in their later life. Further studies are needed to move beyond looking simply at the existence of these risk factors and their relationship to suicide. Instead, we should pay attention to whether age-related life events appear within the sequence because it is important to consider in studying individuals’ later mental health outcomes, including suicide.

## 5. Conclusions

Our study indicates that young adults have faced more suicide risk factors than adolescents in rural China. Strain theory of suicide contributes a new perspective for us to better understand this circumstance and provides some effective ways for rural young adults to lower suicide risks in the society.
